# Non-Arteritic Anterior Ischemic Optic Neuropathy Following COVID-19 Vaccination

**DOI:** 10.3390/vaccines10060931

**Published:** 2022-06-10

**Authors:** Wen-Yun Lin, Jin-Jhe Wang, Chien-Hsiung Lai

**Affiliations:** 1 School of Medicine, College of Medicine, Chang Gung University, Taoyuan 33302, Taiwan; stella980459@gmail.com; 2 Department of Ophthalmology, Chang Gung Memorial Hospital, Chiayi 61363, Taiwan; leo81819@gmail.com; 3 Department of Nursing, Chang Gung University of Science and Technology, Chiayi 61363, Taiwan; 4 School of Traditional Chinese Medicine, College of Medicine, Chang Gung University, Taoyuan 33302, Taiwan

**Keywords:** non-arteritic anterior ischemic optic neuropathy, COVID-19 vaccination, ocular adverse reaction

## Abstract

People are advised to receive a vaccine booster as the Delta and Omicron variants of severe acute respiratory syndrome coronavirus 2 (SARS-CoV-2) emerge, but various adverse ocular reactions after vaccination have been reported. NAION following COVID-19 vaccination appears extremely rarely. Here, we report a case of a 61-year-old female with sudden painless blurred vision developing NAION after receiving ChAdOx1 nCoV-19 (AstraZeneca) vaccination and provide an in-depth explanation of the possible mechanisms regarding the hypercoagulable state after vaccination. Our report adds to the literature on potential adverse ocular effects after COVID-19 vaccination, and we as ophthalmologists recommend that clinicians should increase awareness of this possible ocular complication when evaluating patients with sudden visual disturbance with a recent history of vaccination.

## 1. Introduction

As the Delta and Omicron variants of severe acute respiratory syndrome coronavirus 2 (SARS-CoV-2) emerge, people are advised to receive a vaccine booster [1], but they may have to bear the risk of developing various adverse ocular reactions after vaccination, for instance, uveitis, herpetic eye disease, acute macular neuroretinopathy, optic neuritis, and acute corneal graft rejection [2]. Non-arteritic anterior ischemic optic neuropathy (NAION) has been reported in extremely rare cases; to the best of our knowledge, only one of the six previous cases in the literature is associated with the ChAdOx1 nCoV-19 (AstraZeneca) vaccine [3,4,5,6,7,8]. We as ophthalmologists herein present a case of developing NAION after ChAdOx1 nCoV-19 (AstraZeneca) vaccination to increase awareness of this potential adverse ocular event.

## 2. Case Presentation

**Figure 1:**
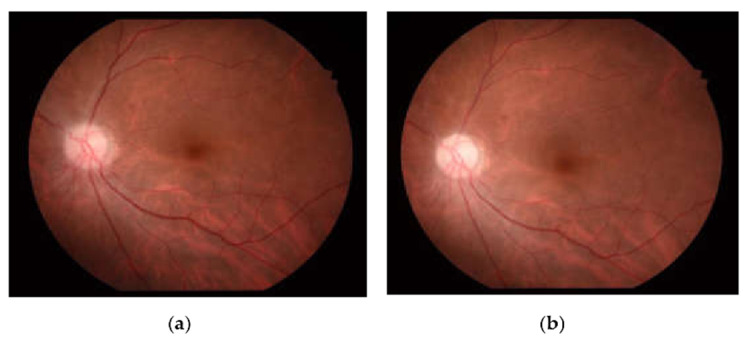
Fundus photography of the left eye (**a**) on initial examination showed pinkish optic disc with optic disc edema (Frisen scale grade 2), and (**b**) after treatment with oral prednisolone, the optic disc edema resolved, and the optic disc became pale with a gliotic appearance in the temporal margin.

**Figure 2:**
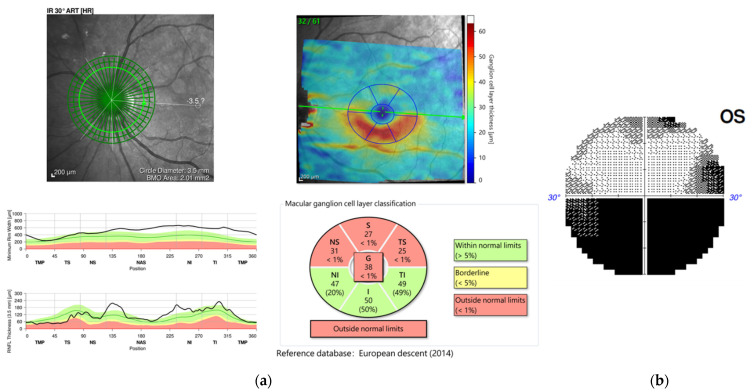
(**a**) Optical coherence tomography (OCT) showed peripapillary retinal nerve fiber layer (pRNFL) edema and thinning of the macular ganglion cell layer (GCL) in the superior hemisphere in the patient’s left eye, (**b**) corresponding to an inferior altitudinal visual defect.

**Figure 3:**
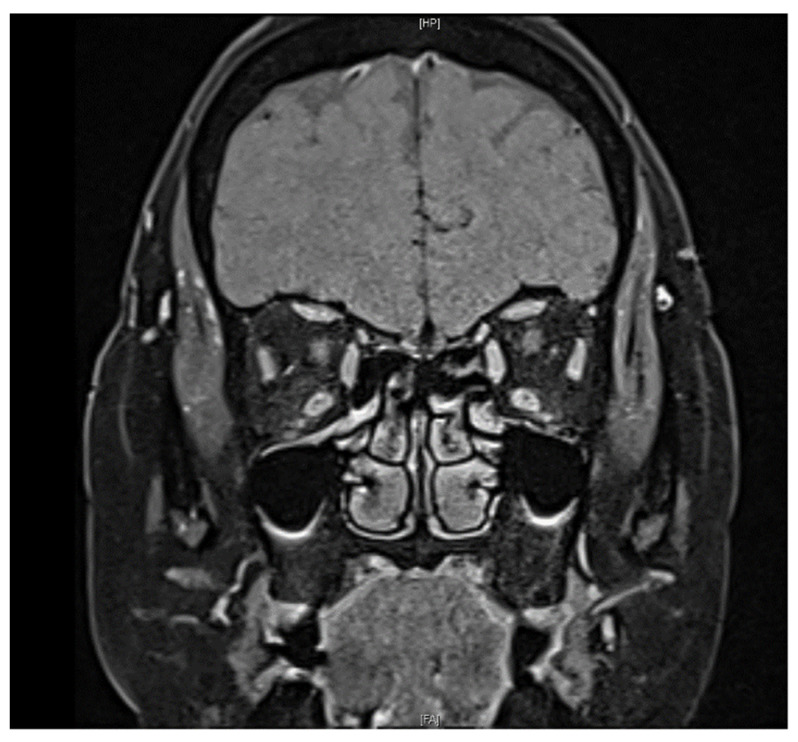
Fat-suppressed T1 coronal image on MRI revealed no contrast enhancement of the optic nerve in both eyes.

A 61-year-old female with a past medical history of controlled hypertension and borderline hyperlipidemia experienced sudden painless blurred vision in the left eye upon awakening. This patient had received the first dose of the ChAdOx1 nCoV-19 (AstraZeneca) vaccine seven days before the onset of her ocular symptoms. She suffered from an initial black shadow in the inferior quadrant with hazy vision upon awakening and had a headache in the temporal region. No other systemic symptoms regarding giant cell arteritis, such as scalp tenderness and jaw claudication, were noticed. She had a history of cataract surgery in both eyes (left eye: a year ago; right eye: seven months ago) without acute vision loss. On ophthalmic examination, her visual acuity was 20/20 and 20/50 in the right and left eye, respectively. No relative afferent papillary defect (RAPD) or color blindness were noted in either eye. Her intraocular pressure was within a normal range (right eye: 12.7 mmHg; left eye: 17.6 mmHg). Fundoscopic examination revealed a normal cup-to-disc ratio in both eyes (0.3/0.3) and pinkish optic disc with clear margin in the right eye, but optic disc edema (Frisen scale grade 2) in the left eye (Figure 1a). Humphrey 30-2 visual field testing identified an inferior altitudinal visual defect in the left eye. Optical coherence tomography (OCT) showed peripapillary retinal nerve fiber layer (pRNFL) edema and thinning of the macular ganglion cell layer (GCL) in the superior hemisphere in the patient’s left eye, corresponding to an inferior altitudinal visual defect (Figure 2). Fluorescein angiography revealed filling delay, decreased choroidal perfusion, and optic disc leakage. Neither obvious edema nor increased contrast medium enhancement of bilateral optic nerves was shown on the MRI of brain (Figure 3), and therefore, the patient was less likely to be a case of optic neuritis. To exclude giant cell arteritis, laboratory studies were examined, including full blood count, erythrocyte sedimentation rate (ESR), and D-dimer, showing normal results. In addition, the antinuclear antibody (ANA) and rapid plasma reagin (RPR) tests were both negative.

**Figure 4:**
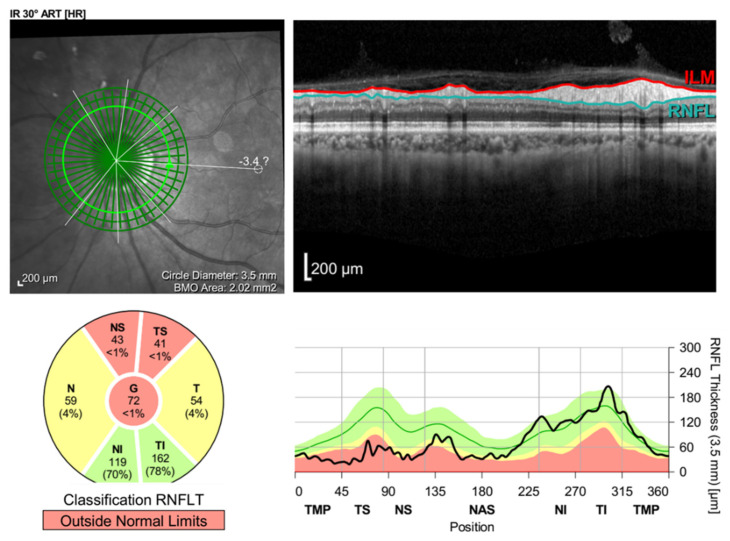
Retinal nerve fiber layer (RNFL) analysis of the left eye revealed RNFL loss after resolution of the optic disc edema.

Therefore, the patient was diagnosed with non-arteritic anterior ischemic optic neuropathy (NAION) in the left eye. She was first offered a two-week course of oral prednisolone 60 mg/day, and then tapered down to 30 mg/day for another two weeks. After a six-week follow-up, her visual acuity became 20/20 in the right eye and 20/80 in the left eye, and the fundoscopic examination revealed the resolution of left optic disc edema (Figure 1b), while the OCT showed diminution of the retinal nerve fiber layer (Figure 4).

## 3. Discussion

NAION following COVID-19 vaccination appears extremely rarely. Among seven reported cases, including this study, all patients, comprising four females and three males, developed NAION unilaterally, and the onset of the ocular symptoms occurred within 9 days after COVID-19 vaccination. Most of the patients were adults aged 50 years or older except for two patients (aged 40 and 46 years) [3,4,5,6,7,8], and they had one or more risk factors for developing NAION, for instance, a small cup-to-disc ratio, diabetes mellitus, and hyperlipidemia. In fact, all previous cases were diabetic patients except for two healthy patients without any systemic or ocular diseases. Our patient did not suffer from diabetes mellitus, the most well-known risk factor for developing NAION [9], but hypertension, hyperlipidemia, and prior cataract surgery (though still controversial as a risk factor) may have predisposed her to NAION. Regarding the treatment, our patient showed the resolution of optic disc edema after administration of oral prednisolone, and this finding was also found in three of the previous reported cases, in which two of the patients even experienced improvement in visual acuity [5,7,8]. 

Even though the exact etiology of NAION remains unclear, an immune complex-mediated vasculopathy has been proposed by Rika Tsukii et al. as a possible mechanism in the development of NAION after COVID-19 vaccination [3]. Kristine Nachbor M.S. et al. and Abid Haseeb et al. assumed a vaccine-induced inflammatory cascade leading to immune-mediated microangiopathy as another possible mechanism [5,6]. In the previous case reported by Kristine Nachbor M.S. et al., a 64-year-old female showed the resolution of the optic disc edema and experienced improvement in her visual acuity after the administration of oral prednisolone [5]. This could be due to the anti-inflammatory effect of the steroid. However, in our patient, though the optic disc edema also resolved after the administration of oral prednisolone, her visual acuity still worsened, and OCT showed the diminution of the retinal nerve fiber layer. This implies that there might be another explanation for the development of NAION after she received COVID-19 vaccination.

Our patient developed NAION seven days after receiving ChAdOx1 nCoV-19 (AstraZeneca) vaccination, but she only had minimal cardiovascular risk factors (controlled hypertension and borderline hyperlipidemia) for NAION. In fact, two previous reported cases of NAION following COVID-19 vaccination also had no cardiovascular risk factors, and the patients even experienced an early-onset NAION (aged 46 and 55 years) [3,8]. This finding is interesting since current studies show that NAION tends to occur in older patients with underlying systemic diseases such as hypertension and diabetes mellitus [9]. With support from previous reported cases of unusual arterial and venous thrombotic events after ChAdOx1 nCoV-19 (AstraZeneca) vaccination [10], we believe that the development of NAION in our patient might be associated with a hypercoagulable state after vaccination. This argument can be further supported by the prospective cohort study conducted by Kuhli-Hattenbach et al. They found a strong association between the onset of NAION at a relatively young age and thrombophilic disorder and noticed a connection between the absence of cardiovascular risk factors in patients with NAION and thrombophilic disorder [11]. This finding is exemplified by three cases of NAION described as an atypical manifestation of COVID-19 diseases. Rho et al. reported a 43-year-old male with underlying systemic diseases of diabetes mellitus and borderline hyperlipidemia presenting NAION two weeks after testing positive for SARS-CoV-2 by nasal swabbing [12]. Additionally, Larrea et al. presented two cases of NAION after COVID-19 disease diagnosis, even though the 39-year-old female and 58-year-old male had no systemic risk factors for NAION [13]. These three patients all met the characteristics of minimal or no cardiovascular risk factors and experienced an early onset of NAION described by Kuhli-Hattenbach et al., and the authors argued that the hypoxemia and hypercoagulability caused by COVID-19 might contribute to the development of NAION [12,13]. As compared with the above three cases, our patient also had minimal cardiovascular risk factors (controlled hypertension and borderline hyperlipidemia) despite developing NAION at an older age of 61 years, and the previous two reported cases of patients who experienced early-onset NAION (aged 46 and 55 years) also met the characteristics of no systemic risk factors described by Kuhli-Hattenbach et al. [3,8]. Therefore, our finding is in agreement with the argument proposed by Kuhli-Hattenbach et al., i.e., that the development of NAION might be linked to a hypercoagulable state. 

## 4. Conclusions

In conclusion, we describe a case of NAION developed following ChAdOx1 nCoV-19 (AstraZeneca) vaccination and provide in-depth explanation of the possible mechanisms. Our report adds to the literature on potential adverse ocular effects after COVID-19 vaccination. We therefore recommend that ophthalmologists should increase the awareness of this possible ocular complication when evaluating patients with sudden visual disturbance with a recent history of vaccination.

## Data Availability

The data presented in this study are available upon request from the corresponding authors.
